# Illness trajectories of incurable solid cancers

**DOI:** 10.1136/bmj-2023-076625

**Published:** 2024-03-01

**Authors:** Eric C T Geijteman, Evelien J M Kuip, Jannie Oskam, Diana Lees, Eduardo Bruera

**Affiliations:** 1 Department of Medical Oncology, Erasmus MC Cancer Institute, Rotterdam, The Netherlands; 2 Department of Medical Oncology and Department of Anesthesiology, Pain and Palliative Care, Radboudumc, Nijmegen, The Netherlands; 3 Patient author, The Netherlands; 4 Department of Respiratory Medicine, Liverpool University Foundation Teaching Hospital, United Kingdom; 5 Department of Palliative Rehabilitation and Integrative Medicine, University of Texas MD Anderson Cancer Center, Houston, USA

**What you need to know**

- New systemic anticancer treatments, such as targeted therapy and immunotherapy, have made treating incurable solid cancer more successful but also more challenging and unpredictable
- Updated treatment illness trajectories for patients with incurable solid cancer include major temporary improvement, long term ongoing response, and rapid decline
- Discuss with the patients and caregivers their goals and preferences, ideally at the time of diagnosis, particularly because of the prognostic uncertainty associated with systemic anticancer treatment
- Supportive and palliative care should be provided in conjunction with newer anticancer therapies to address patients’ physical, psychological, social, and spiritual challenges

Cancer remains one of the leading causes of death worldwide,^1^ and the number of cancer related deaths is expected to be over 16 million by 2040.^2^ Over 90% of all new cases of cancer are solid cancer types, including breast, lung, colorectal, and prostate cancer.^3^ When solid cancer is incurable, patients often have a life expectancy of less than one year. In this context, palliative anticancer treatment can maintain or improve quality of life and increase life expectancy.

Systemic anticancer treatment (SACT) is the most widely used palliative care treatment for incurable solid cancer. SACT offers the advantage of decreasing disease burden by targeting cancer cells throughout the body compared with locally targeted surgery or radiation. Previously, the only SACT licensed to treat solid incurable cancers were hormonal and chemotherapies, and a patient’s trajectory after diagnosis was relatively predictable until death, whereby they would experience a decline in physical health over a period of weeks to months.^4^ In the past 25 years, two other SACT modalities, immunotherapy and targeted therapy (drugs to target specific genes and proteins), were introduced that have extended the life expectancy of patients with solid incurable cancers, allowing more time for patients, their care givers, and healthcare systems to anticipate palliative needs and plan end of life care.

In this article, we build on an earlier review of the typical trajectories of patients with life threating illnesses^4^ and present revised illness trajectories for patients with incurable solid cancer. We also give an overview of important current challenges and highlight the role of supportive and palliative care in tackling these challenges.

## Types of illness trajectories

Illness trajectories are used to describe the course or progression of a disease as experienced by a patient over time. Reflecting on timeframes and probable future needs of the patient helps multidisciplinary teams organise appropriate care in different phases of disease, as well as towards death.^4^ In addition, knowledge and information about the illness trajectories can give patients and their care givers a sense of control.^4^ After our review of the evidence, we outlined types of SACT, and table 1 gives examples of their use in eight incurable solid cancers. Below, we update the illness trajectories of patients with incurable solid cancers.

**Table 1:** Types of SACT and examples of incurable solid cancer types

Types of therapy	Cancer types	Target for therapy	Indicators for response	Response rate*	Median time to response	Median duration of response
**Hormonal therapy**						
Anti-oestrogens, aromatase inhibitors	Breast cancer^5^-^7^	Oestrogen receptor and/or progesterone receptor expression	—	~30-50%	Within weeks	Months to years
LHRH agonists, androgen receptor blockers, androgen synthesis inhibitors	Prostate cancer^8^-^10^	—	—	~50-70%	Within weeks	Months to years
**Chemotherapy**						
Alkylating agents, antimetabolites, anti-tumour antibiotics, topoisomerase inhibitors, mitotic inhibitors	Pancreatic cancer^11^	—	—	30-40%	Within weeks	Several months
	Colorectal cancer^12^ ^13^	—	—	~50%		
	Biliary tract cancer^14^	—	—	~20-30%		
**Targeted therapy (drugs to target specific genes and proteins)**						
TKI	Gastrointestinal stroma cell tumour^15^ ^16^	—	KIT exon 11 mutation	≤85%	Within days to weeks	Up to years
BRAF/MEK inhibition	Melanoma^17^ ^18^	BRAF V600 mutation	—	≤90%	Within days to weeks	Weeks to years
TKI	Non-small cell lung cancer^19^-^21^	ALK rearrangement, ROS 1 rearrangement, EGFR mutation	—	≤60-80%	Within days to weeks	Weeks to years
**Immunotherapy**						
PD-1 antibodies, CTLA-4 antibodies	Melanoma^22^	—	—	~60%	Within months up until 1 year	Many years
PD-1 antibodies, CTLA-4 antibodies	Non-small cell lung cancer^23^ ^24^	—	PD-L1 expression	~40-50%	Within months	Months to years
PD-1 antibodies	Colorectal cancer^25^	MSI		~40-50%	Within months	Months to years

* Percentage of patients who achieve a response, including complete response (complete disappearance of lesions) or partial response (reduction in the sum of maximal tumour diameters by ≥30%). Many of the hormonal and chemotherapies may also stabilise an advanced cancer, which may be of great importance for patients in order to increase life expectancy.

ALK=anaplastic lymphoma kinase; BRAF=v-Raf murine sarcoma viral oncogene homologue B1; CTLA=cytotoxic T lymphocyte antigen; EGFR=epidermal growth factor receptor; KIT=receptor tyrosine kinase type III; LHRH=Luteinising hormone-releasing hormone; MEK=mitogen-activated protein kinase kinase; MSI=Microsatellite instability; PD=Programmed death; PD-L=Programmed death ligand; ROS=Proto-oncogene tyrosine-protein kinase; TKI=Tyrosine kinase inhibitor.

### Traditional illness trajectory

The traditional illness trajectory for patients with incurable solid cancer in 2005 was steady progression from diagnosis until death.^4^ This trajectory typically still applies to patients who do not receive anticancer treatment or those who receive palliative hormonal therapy or palliative chemotherapy (box 1).

**Fig 1:**
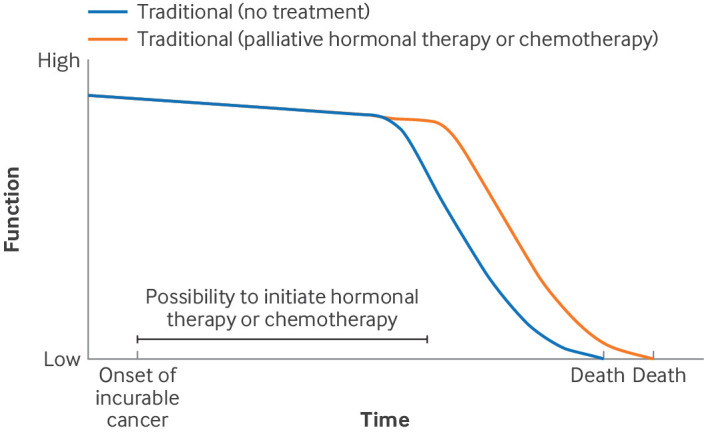
Patients within the traditional disease trajectory gradually deteriorate and then enter a short period of evident decline ending in death. Compared with receiving no treatment, patients treated with chemotherapy or hormonal therapy may live months to years longer

Box 1 **Patient vignette illustrating the traditional illness trajectory (fig 1)**

A 72 year old widow complained of painless jaundice. She was diagnosed with pancreatic cancer with liver metastases and malignant biliary obstruction. After stent insertion, chemotherapy (oxaliplatin, irinotecan, fluorouracil, and leucovorin (FOLFIRINOX)) was started. In addition, palliative home care and support was offered from a spiritual counsellor. After eight cycles of chemotherapy, during which her condition was deteriorating, there was a progression of her metastatic cancer. To provide more appropriate care, the chemotherapy was stopped, and she was transferred to a hospice, dying several weeks later, six months after initial diagnosis.

To emphasise the possibility to temporarily stabilise the advanced cancer by suppressing disease progression with palliative hormonal therapy or palliative chemotherapy, unlike the original 2005 figure, we make a distinction between patients with such therapy and those without. For some patients being treated with hormonal therapy (such as those with prostate or breast cancer), it may take years before the cancer becomes resistant to treatment. This differs from patients receiving chemotherapy, whether monotherapy or with multiple agents, which aims to prolong life by a few months usually.

In recent decades, improvement has been made by the advent of newer, effective SACT modalities, including chemotherapies, and sequencing regimens. Some of these may be initiated when the disease has progressed after a previous treatment, earlier in the disease course.

### Major temporary improvement

This illness trajectory applies to patients who are treated with targeted anticancer therapy, where genes and proteins that directly affect cancer cells are targeted.^26^ ^27^ ^28^ Most targeted therapies are useful only in cases of specific genetic mutation or rearrangement. Response to treatment may be within days, and response rates may be up to 90%.^19^ In this scenario, therapy is often used in patients who are eligible to receive the treatment, but also select cohorts of patients who are seriously ill and near to death at time of diagnosis (box 2). The main disadvantage of targeted therapies is that some may cause intolerable adverse events, and all therapies eventually lose efficacy as tumours develop resistance and cancers relapse.^28^

**Fig 2:**
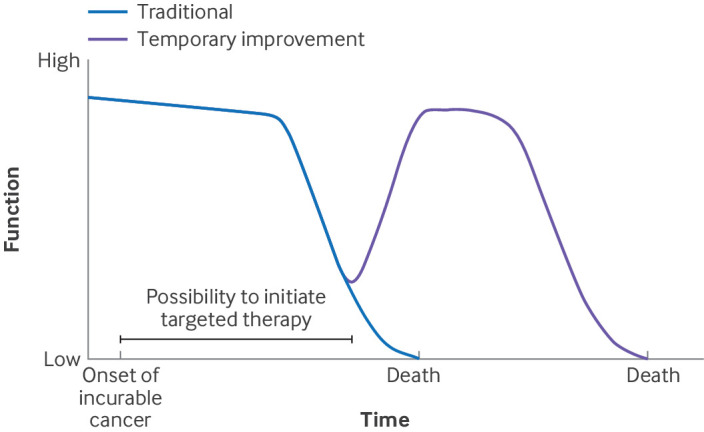
Major temporary improvement: patients who benefit from targeted therapy (drugs to target specific genes and proteins) may respond to such a degree that their condition improves for weeks or even years before their final deterioration

Box 2 **Example of an illness trajectory with major temporary improvement (fig 2)**

A 58 year old woman complained of severe hip pain and progressive dyspnoea. She was diagnosed with stage 4 non-small cell lung cancer with metastases in lymph nodes and bones. The hip pain was a result of a bone metastasis and was treated with high doses of fentanyl and naproxen. Her dyspnoea increased following the development of a bacterial pneumonia, and she was transferred to intensive care, where broad spectrum antibiotics and artificial ventilation were started. As the patient had proto-oncogene tyrosine-protein kinase 1 rearrangement, treatment with crizotinib, a targeted therapy, was also started. Within a week, the dyspnoea subsided, together with her need for artificial ventilation. The patient’s hip pain was also greatly reduced, and thus the high doses of opioids could be reduced. Several days after admission to intensive care, she was discharged in good physical condition.

An example of incurable cancer with genetic mutations and rearrangements is epidermal growth factor receptor (EGFR) mutation in non-small cell lung cancer. In the early 2000s, the median overall survival of patients with incurable non-small cell lung cancer with palliative chemotherapy was 14-16 months.^29^ A randomised controlled trial of 556 patients with previously untreated EGFR mutation-positive advanced non-small cell lung cancer demonstrated a response rate of 80%, and median duration of response of 17.2 months.^21^

### Long term ongoing response

Immunotherapy enhances a patient’s immune system to fight cancer^26^ ^30^ and includes the use of immune checkpoint inhibitors (such as ipilimumab, nivolumab, and pembrolizumab), vaccines, and monoclonal antibodies. Through the introduction of immunotherapy, patients with an incurable solid cancer may live much longer (box 3). Even after the discontinuation of immune checkpoint inhibitors, patients can enter durable remission because of initial response to immunotherapy, leading to a disease control over many years.

**Fig 3:**
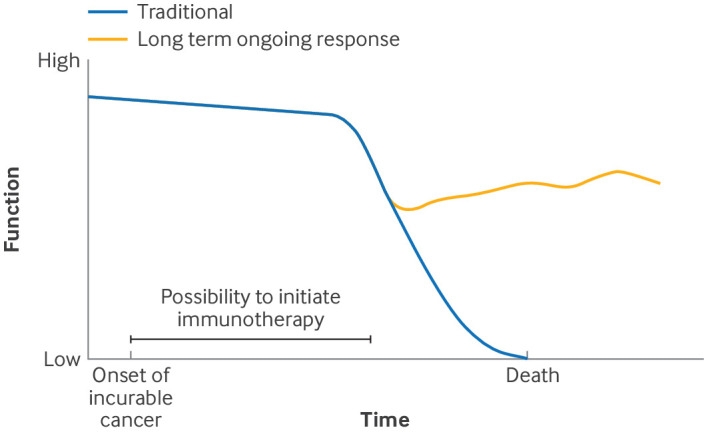
Long term ongoing response. Patients may enter long term, durable remission and receive follow-up care over a longer period

Box 3 **Example of an illness trajectory with a long term response to therapy (fig 3)**

A 49 year old man was diagnosed with metastatic melanoma, with metastases to the lungs, bones, and liver, five years after a resection of a melanoma supraclavicular. Initially, he was treated with combination therapy of nivolumab and ipilimumab. After four cycles of the combination therapy, computed tomography (CT) showed a good response to treatment. Apart from developing facial vitiligo and arthralgia, due to an autoimmune response to melanocytes and joints, respectively, he had no other adverse events. After these four cycles of combination therapy, immunotherapy treatment was continued with monotherapy of nivolumab, which continued until he was finally treated for two years with immunotherapy. Three years later he was still in good physical health, besides having chronic arthralgia. A CT scan showed no signs of any illness returning. Since he was first diagnosed, he struggled to get his life back on track, and, directly after the diagnosis of metastatic melanoma, he was declared medically unfit for work for the rest of his working life.

In the case of advanced melanoma, about 15 years ago, before the introduction of immunotherapy, the average survival for patients was six to nine months.^31^ In one recent, large randomised controlled trial, the overall response rate of patients treated with combination therapy of two different immune checkpoint inhibitors at 6.5 years was 58%, in which the median duration of response was not reached, suggesting durable clinical benefit.^22^ Currently, it remains unknown which patients with metastatic melanoma are likely to respond to treatment with immunotherapy. For some cancers, such as colon cancer and lung cancer, understanding the patient’s genomic profile may enable a more accurate prediction of successful treatment, and facilitate a personalised therapeutic approach. A disadvantage of the therapy is that it may take some time, from months up to a year, to achieve maximum effect.^32^

### Rapid decline due to adverse event

A disadvantage of the different SACTs is the occurrence of adverse events, as almost all patients receiving chemotherapy experience one or more side effects such as nausea and vomiting, diarrhoea, and neuropathy.^33^ In a minority of patients, adverse events may be so severe that some patients die earlier than expected (box 4).

**Fig 4:**
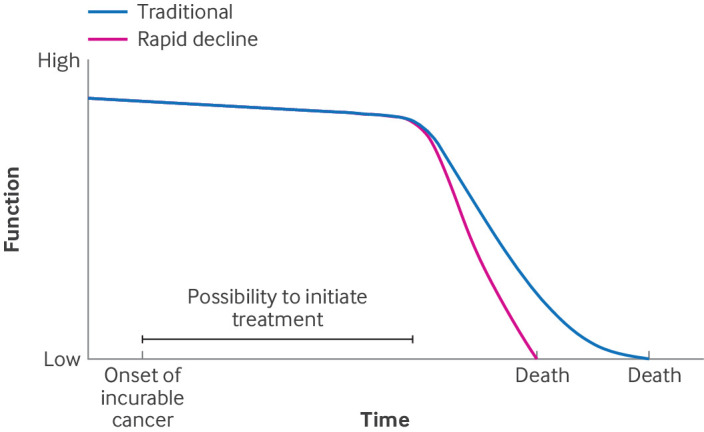
Rapid decline due to adverse event. Patients may die prematurely due to adverse events

Box 4 **Example of an illness trajectory with rapid decline of function due to adverse event (fig 4)**

A 56 year old man, had been diagnosed with renal cell carcinoma with pulmonary metastases. Without SACT, overall survival of these patients is about six months.^34^ A combination therapy of three-weekly ipilimumab and nivolumab was started. A few days after the third cycle, the patient developed complaints of left flank pain with fever and general weakness. Blood tests showed thrombocytopenia, markedly elevated ferritin, and liver enzymes. A CT scan showed the disease was stable, but had developed substantial splenomegaly. With the working diagnosis of immune mediated haemophagocytic syndrome, high dose prednisolone was started and later intravenous immunoglobulin and tocilizumab. Despite these immunosuppressive therapies, the patient’s condition deteriorated, and he died in hospital three weeks after onset of symptoms.

Rates of adverse events differ according to SACT therapies. For example, adverse events secondary to hormonal therapy are due to unwanted changes to normal hormone physiology. These may include complaints of hot flashes, sweating, and tiredness, which can substantially affect the patient’s qualify of life. Adverse events related to targeted therapy are diverse, but severity is usually low, particularly compared with other SACT modalities.^35^ Low grade toxicity can also cause significant deterioration in the quality of life of patients, ultimately necessitating a reduction in the dose or even discontinuation.

Side effects of immunotherapy regimens may involve any organ system, but the thyroid, colon, liver, and lung are particularly prone to adverse events.^36^ Severe immune related adverse events are reported in 50% and more of patients treated with a combined immune checkpoint inhibitor.^22^ In some patients, side effects can persist for years after discontinuing immunotherapy. In a review study of immune toxicities of immune checkpoint inhibitors it was highlighted that chronic immune related adverse events affected up to 40% of patients after stopping immunotherapy.^37^ Fatal adverse events occur in 0.4-1.2% of patients.^37^

## What are the limitations in interpreting illness trajectories?

Although illness trajectories may give guidance to patients, care givers, and healthcare professionals in the anticipated medical course for an individual at the point of diagnosis, there are limitations for their use. The illness trajectories presented underline that the prognosis of patients with incurable solid cancers is unpredictable: some patients will benefit greatly, and others may not at all. Many patients with incurable solid cancer still die within months of being diagnosed, others may live for a considerable time.^22^ A retrospective study in the Netherlands that included almost two million patients with solid cancer showed a median survival of less than 7 months in patients with incurable solid cancer.^38^ Research investigating the actual increase of survival rates within cancer care has been limited to specific cancer types, and with specific genetic mutations or rearrangements (table 1).^19^

Applying one illness trajectory can oversimplify a single patient’s journey, as they may experience multiple trajectories. This may be particularly relevant for patients who are treated with different combined and consecutive anticancer therapies.^39^ The different illness trajectories are applicable on a population level but may evolve atypically for individual patients. Therefore, there are practical challenges to using illness trajectories in individual care planning. These challenges may be greater when caring for older patients or those who have concurrent multiple, life limiting comorbidities.^32^

Immunotherapy and targeted therapy may cost up to £100 000 (€110 000) a year per patient.^19^ ^40^ In high income countries, more than half of all incurable solid cancers can be treated with immunotherapy or targeted therapy,^19^ ^30^ whereas patients from most low and middle income countries do not have access to these treatments, and so the illness trajectories linked to these therapies are largely irrelevant.^41^

## What are the current key challenges?

Although current SACT options for patients with an incurable solid cancer have made it possible for patients to live longer with a good quality of life, new challenges have emerged for patients, their care givers, and for healthcare professionals.

### Psychological challenges

Probably the most important psychological challenge that has arisen as a result of SACT is patients’ fear and uncertainty with respect to treatment and prognosis. Often the result of the different directions a patient’s illness trajectory may take at various times, fear and uncertainty can be debilitating and overwhelming for patients and their care givers,^42^ ^43^ ^44^ and are associated with poorer quality of life and worse psychosocial health.^45^ Initially, there may be fear and uncertainty about whether the SACT will result in the intended effect. If the patient responds to treatment, the next question is usually if and when the cancer will progress.^46^ In our experience, this usually arises from patients treated with targeted therapy, when the cancer will eventually relapse due to resistance, but can also occur in patients treated with other SACTs.

### Social and spiritual challenges

Patients can experience numerous social and spiritual challenges after receiving a diagnosis of incurable solid cancer. Patients may need to find a new balance and perspective regarding loss of life,^42^ especially in the early, often more uncertain, phase. In our experience, patients find it important to continue with things in life that they enjoy as much as possible. For example, about half of patients with advanced cancer are of working age,^47^ and when diagnosed they are often advised to stop working immediately (as seen in the example of box 4). Given the improvements in cancer care, continuing work may be advisable, by increasing quality of life through the continued sense of normalcy, daily structure, and social belonging.^47^ ^48^

### Physical challenges

Physical symptoms have a major impact for patients with incurable solid cancer. In a systematic review of symptoms including 44 studies and more than 25 000 patients several symptoms, such as fatigue, lack of energy, pain, and weakness, occur in more than half of the patients.^49^ The frequency and severity of physical symptoms may increase during SACT, and inadequate symptom control can negatively affects quality of life. Conversely, SACT may decrease tumour size and activity, leading to improvement. Specifically, the introduction of immunotherapy has led to different, often chronic, physical challenges due to immune related adverse events which can be difficult to treat.^37^

### Too much care?

Because of the unpredictable prognosis of patients with incurable solid cancers, care towards the end of life may be too aggressive, including the use of newer SACT modalities, hospitalisation, or intensive care unit admission. A retrospective health record cohort study from 280 US cancer clinics including patients diagnosed with cancer who received treatment and died within four years of diagnosis showed that the total use of SACT within 30 days of death did not change between 2015 and 2019 (about 39% of all patients received SACT within 30 days of death).^50^ However, the use of chemotherapy decreased (from 26% in 2015 to 16% in 2019), while the use of immunotherapy increased (from 5% in 2015 to 18% in 2019).^50^ Aggressive treatment in the last days and weeks of life is associated with decreased quality of life, prolonged bereavement, and increased healthcare costs.^51^ ^52^

Counselling patients at the end of life on prognosis, time of death, or level of anticipated recovery remains difficult, in part due to the unpredictability of individual response to SACT therapies. This is particularly relevant for patients treated with targeted therapy, who may respond quickly to therapies. For patients treated with immunotherapy, it can be difficult to determine whether a patient’s functional decline is the beginning of their imminent death or that of a clinical response.

Timely discussion with patients and their care givers about their individual goals and preferences is therefore important.^53^ ^54^ Patients seem to be less willing to undergo invasive treatments than healthcare professionals think.^55^ ^56^ Examples of such communication concepts to promote patient-centred care are advance care planning, shared decision making, and serious illness conversations.^57^ ^58^ ^59^

## What is the role of supportive and palliative care?

**Fig 5:**
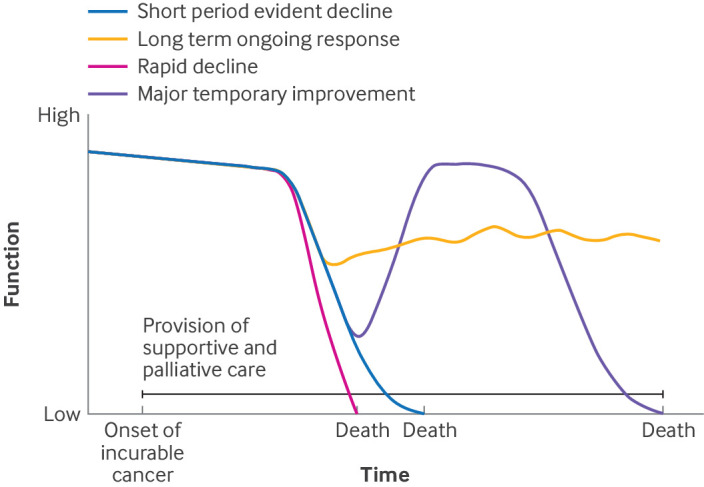
Provision of supportive and palliative care in current cancer care

Multiple trials have confirmed the benefits of early supportive and palliative care alongside routine care for patients with advanced cancer.^56^ ^57^ ^60^ ^61^ It improves quality of life, reduces the burden of symptoms,^57^ ^58^ ^60^ ^61^ and can lead to longer survival.^56^ Examples of supportive and palliative care are providing adequate treatment for physical and psychological symptoms associated with the cancer, while offering additional education and information about the disease and prognosis. In current practice, however, such care is rarely provided, or is given late, in the advanced cancer trajectory.^62^ ^63^ We recommend supportive and palliative care to be provided at time of diagnosis for every patient with advanced cancer (fig 5).^64^ ^65^ ^66^ ^67^

**Education in practice**

- How do you discuss with patients with incurable solid cancer their goals and preferences in the short and long term?
- Think about the last time you treated a patient with solid incurable cancer. Reflect on the challenges you encountered during this treatment. What changes, if any, might you make to this treatment next time?
- What barriers do you perceive in applying the updated treatment illness trajectories in your clinical practice?

**How this article was made**

Illness trajectories are concepts that have been developed previously,^4^ and we updated the trajectory of patients with incurable cancer following review of the evidence base and in line with our experiences in clinical practice. To do this, we reviewed landmark trials, observational studies, and reviews that investigated eight types of incurable solid cancer. Additional references used in this article were identified from our personal archives and with the help of experts we approached. In addition, we systematically searched the Cochrane Library, Embase, and Medline, as outlined below.

- Medline: (((Terminal Care / OR Terminally Ill / OR Palliative Care /) AND exp Neoplasms /) OR ((incurab* OR noncurab* OR non-curab* OR terminal* OR end-of-life) ADJ3 (cancer* OR tumo*)).ab,ti,kw.) AND (* Immunotherapy / OR * Immunotherapy, Active / OR * Immunotherapy, Adoptive / OR * Molecular Targeted Therapy / OR (immunother* OR immun*-ther* OR (target* ADJ3 therap*)).ti.) AND english.la. NOT (exp animals/ NOT humans/)
- Embase: ('incurable cancer'/exp OR 'cancer palliative therapy'/de OR (('incurable disease'/exp OR 'terminal care'/de OR 'terminally ill patient'/de OR 'palliative therapy'/de) AND 'neoplasm'/exp) OR ((incurab* OR noncurab* OR non-curab* OR terminal* OR end-of-life) NEAR/3 (cancer* OR tumo*)):ab,ti,kw) AND (immunotherapy/mj/de OR 'active immunotherapy'/mj/de OR 'adoptive immunotherapy'/mj/exp OR 'antibody therapy'/mj/exp OR 'cancer immunotherapy'/mj/exp OR 'molecularly targeted therapy'/mj/de OR (immunother* OR immun*-ther* OR (target* NEAR/3 therap*)):Ti) NOT [conference abstract]/lim AND [english]/lim NOT ([animals]/lim NOT [humans]/lim)
- Cochrane: (((incurab* OR noncurab* OR non-curab* OR terminal* OR end-of-life) NEAR/3 (cancer* OR tumo*)):ab,ti,kw) AND ((immunother* OR immun* NEXT ther* OR (target* NEAR/3 therap*)):Ti)

**How patients were involved in the creation of this article?**

Coauthor Jannie Oskam is a patient. She was diagnosed with metastatic breast cancer in 2019 and wrote the book *Tussenland* (which could be freely translated as “the place in between”) in Dutch about her, and others’, experiences as a patient with incurable solid cancer. We also asked Renée Dubois, patient advocate of the patient organisation Lung Cancer Netherlands and living with stage 4 ALK-positive lung carcinoma, to comment the developing manuscript. Both read all drafts of the article and provided input particularly related to key challenges regarding implementation. They also helped develop the education in practice questions.

**Questions for future research**

- What is the effect of integrating the updated treatment illness trajectories at an individual level, and how best can we measure these effects?
- How can healthcare professionals effectively communicate prognostic uncertainty to patients and their caregivers?

**Additional educational resources**

- Information on the Serious Illness Program, including the Serious Illness Conversation Guide which may serve as a framework for healthcare professionals to explore what is most important to patients with incurable solid cancer. https://www.ariadnelabs.org/serious-illness-care/
- Information on the management of different patient problems and their management can be obtained from the following:
  - ESMO. Clinical Practice Guidelines: Supportive and palliative care. https://www.esmo.org/guidelines/guidelines-by-topic/supportive-and-palliative-care
  - ASCO. Clinical care initiatives. https://old-prod.asco.org/news-initiatives/current-initiatives/cancer-care-initiatives/palliative- care-oncology

**Information resources for patients**

- Ariadne Labs (https://www.ariadnelabs.org/serious-illness-care/)—Helps patients with incurable solid cancer get ready to talk with their healthcare professionals about what is most important to them
- European Society for Medical Oncology (https://www.esmo.org/for-patients/patient-guides)—Guides for patients to better understand their cancer
- American Society of Clinical Oncology (https://old-prod.asco.org/news-initiatives/current-initiatives/cancer-care-initiatives/palliative-care-oncology)—Information on palliative care for patients and their caregivers
- National Cancer Institute (https://www.cancer.gov/about-cancer/advanced-cancer)—Guidance for patients with incurable cancer

## Notes

Patient consent: Not required.

Provenance and peer review: Commissioned, based on an idea from the author; externally peer reviewed.
